# Pembrolizumab versus paclitaxel for previously treated, advanced gastro-esophageal junction cancer: A systematic review and meta-analysis of randomized clinical trials

**DOI:** 10.1097/MD.0000000000031940

**Published:** 2022-12-02

**Authors:** Sarya Swed, Nour Shaheen, Wael Hafez, Nesreen Elsayed Talat, Samah S. Rozan, Rehab Diab, Abdulqadir J. Nashwan, Karam R. Motawea, Hidar Alibrahim, Mhd Kutaiba Albuni, Elias Battikh, Bisher Sawaf, Sheikh Shoib

**Affiliations:** a Faculty of Medicine, Aleppo University, Aleppo, Syria; b Faculty of Medicine, Alexandria University, Alexandria, Egypt; c NMC Royal Hospital, 16th Street, Khalifa City, Abu Dhabi, UAE; d Medical Research Division, Department of Internal Medicine, The National Research Centre, Cairo, Egypt; e Faculty of Medicine, Al-Azhar University, Cairo, Egypt; f Nursing Department, Hamad Medical Corporation, Doha, Qatar; g Department of Internal Medicine, Hamad Medical Corporation, Doha, Qatar; h Department of Psychiatry, Jawahar Lal Nehru Memorial Hospital, Srinagar, Kashmir, India.

**Keywords:** advanced gastro-esophageal junction cancer, meta-analysis, paclitaxel, pembrolizumab

## Abstract

This paper aims to compare the effectiveness and safety of pembrolizumab and paclitaxel as a second line for patients with locally advanced gastroesophageal cancer.

**Methods:**

By searching PubMed, Scopus, Web of Science, and Ovid, any randomized clinical study comparing the effectiveness of paclitaxel and pembrolizumab as second-line therapy for advanced gastroesophageal cancer met the inclusion criteria. Only 3 of the 23 eligible studies that were fully reviewed were eligible for meta-analysis.

**Results:**

The total number of patients included in the meta-analysis was 635 in the pembrolizumab group and 596 in the paclitaxel group. In terms of objective response rate, there was no statistically significant difference between pembrolizumab and paclitaxel (relative risk = 1.10, 95% CI = 0.80–1.50, *P* = .57). Furthermore, Pembrolizumab and paclitaxel did not differ in terms of the rate of partial response statistically significantly from one another, according to the overall analysis (relative risk = 0.93, 95% CI = 0.57–1.52, *P*-value = .78).

**Conclusion:**

There is no difference between pembrolizumab and paclitaxel in objective response rate. The objective response rate shows that doctors may consider either treatment for patients with advanced gastroesophageal cancer, given the time to response is comparable across therapies.

## 1. Introduction

Cancer killed 10 million people in 2020, according to the World Health Organization.^[1]^ Among these deaths, 1 million and ninety thousand were caused by gastric cancer, which includes gastroesophageal cancer, which represents the fifth most common and fourth deadly cancer worldwide in 2020.^[2]^ Esophageal cancer often develops in the cells lining the interior of the esophagus. Anywhere along the esophagus might be the site of esophageal cancer. Men are more likely than women to get esophageal cancer. The rate of occurrence varies by geographic region. Tobacco and alcohol use, certain dietary habits, and obesity may contribute to a greater risk of esophageal cancer in various areas.^[2]^ There is a greater probability of recovery when esophageal cancer is discovered extremely early. Many cases of gastric cancer progress to advanced stages, which are better to be treated by chemotherapy than best supportive care, even in patients with unresectable or metastatic advanced gastric cancer, as it improves the overall survival (OS) and quality of life.^[8]^ The first line of treatment chemotherapy is platin-fluoropyrimidine,^[9]^ which has some limitations in preventing the progression as up to 20% to 35% of cases progress at the first evaluation appointment^[10]^ so, here comes the importance of the second line, which includes the paclitaxel and pembrolizumab in delaying the progression and improving the overall survival.^[11]^ Pembrolizumab is a humanized monoclonal antibody that targets programmed death 1 (PD-1) receptors preventing its interaction with its ligands PD-L1 and PD-L2 that inhibit the suppression of immune response facilitating its antitumor action.^[12]^ This systematic review and meta-analysis of the published literature on pembrolizumab and paclitaxel as a second line for treating advanced gastroesophageal cancer summarizes the evidence published and helps physicians in decision-making during their daily clinical care. Cancer killed 10 million people in 2020, according to the World Health Organization.^[1]^

## 2. Methods

### 2.1. Search strategy and study selection

**Figure 1.**
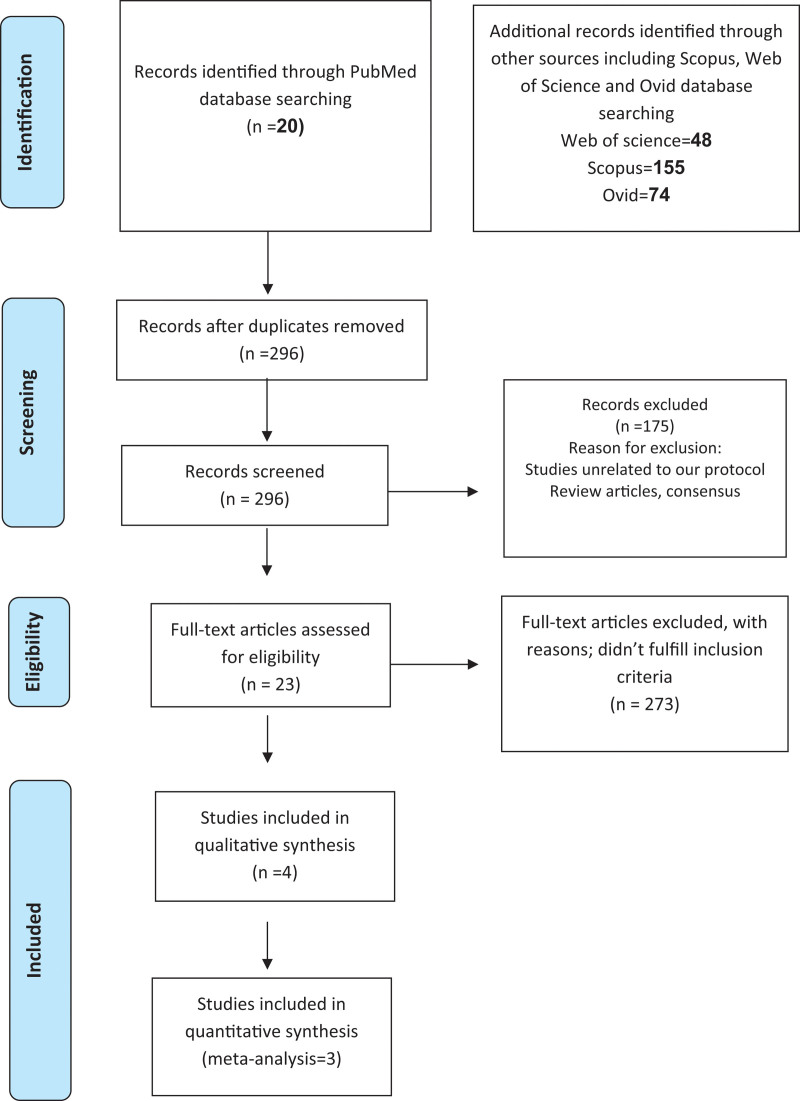
PRISMA Flow diagram of included studies.

The electronic databases PubMed, Scopus, Web of Science, and Ovid database were searched on 20 April 2022., using the terms ((“Pembrolizumab “[Mesh]) AND ((“paclitaxel *”[Mesh]) AND ((“gastroesophageal*”[Mesh]) AND ((“cancer*”[Mesh]). In total, 297 studies were found in PubMed, Ovid, Web of Science, and Scopus using our search criteria. After excluding duplicate studies, studies missing clinical data, review articles, and articles unrelated to our study objective, 296 full-text literature were reviewed. The review included 3 studies that met our inclusion criteria; therefore, 2 studies about vertebral artery dissection after birth were reviewed and analyzed (Fig. 1). In addition, we have uploaded the search strategy for each data base as supplementary material 1, Supplemental Digital Content, http://links.lww.com/MD/H983.

### 2.2. Data extraction

First, all titles and abstracts of papers retrieved by the search strategy were screened for relevance; those that were irrelevant were discarded. The full text was downloaded if the result was relevant. As a second step, 2 review team members independently evaluated the studies’ eligibility using predefined inclusion and exclusion criteria. The reviewers discussed whether or not to include a particular study if they disagreed.

### 2.3. Risk of bias (Quality) assessment

According to the Cochrane risk of bias tool, we evaluated the following bias domains for each RCT(1); selection bias, performance bias, detection bias, and Reporting bias.

### 2.4. Statistical analysis

The effectiveness of Pembrolizumab Versus Paclitaxel for Previously Treated Advanced Gastro-Esophageal Junction Cancer was compared by meta-analysis. For the statistical analysis, we utilized RevMan 5.4. The continuous outcomes were quantified with a 95% confidence interval as mean difference (MD) and standard deviation (SD). A random-effect model was employed if there was heterogeneity (Chi-square *P* value .05); otherwise, a fixed-effect model was utilized. The findings were considered statistically significant if the *P*-value was lower than .05.

## 3. Results

### 3.1. Literature search

Following the search, 267 were rescued. Using Rayyan, one duplication was removed. After removing 175 irrelevant records from our screening of 296 titles and abstracts and 23 full-text publications, we included 4 studies in our systematic review. However, there are just 3 studies included in the meta-analysis. A PRISMA flow chart illustrates the selection process (Fig. 1).

### 3.2. Baseline characteristics

We included 3 randomized clinical trials with a total of 1231 patients: 635 in the pembrolizumab group and 596 in the Paclitaxel group. The frequency and percentage of comorbidities, including hypothyroidism, hyperthyroidism, pneumonitis, infusion reactions, hepatitis, etc., are demonstrated in Table 1. Procedure characteristics of the included studies, including the number of patients, age, sex, other baseline diseases, and follow-up duration, are demonstrated in Table 2.

**Table 1:** Summary of the included studies.

	Charles S. Fuchs (2021)		Hyun Cheol Chung, MD, PhD et al (2021)		Kohei Shitara et al (2018)	
	Pembrolizumab (n = 294)	Paclitaxel (n = 276)	Pembrolizumab (n = 47)	Paclitaxel (n = 44)	Pembrolizumab (n = 294)	Paclitaxel (n = 276)
Related to treatment						
Age	157 (53.4)	233 (84.4)	46 (98)	43 (98)	155 (53%)	232 (84%)
Occurring in ≥ 10% in either group						
Fatigue	35 (11.9)	64 (23.2)	6 (13)	5 (11)	35 (12%)	64 (23%)
Decreased appetite	24 (8.2)	43 (15.6)	1 (2)	11 (25)	24 (8%)	43 (16%)
Nausea	17 (5.8)	50 (18.1)	2 (4)	5 (11)	17 (6%)	50 (18%)
Diarrhea	16 (5.4)	38 (13.8)			16 (5%)	38 (14%)
Anemia	10 (3.4)	41 (14.9)	1 (2)	8 (18)	10 (3%)	39 (14%)
Alopecia	1 (0.3)	111 (40.2)	1 (2)	21 (48)	1 (<1%)	111 (40%)
Neuropathy, peripheral	1 (0.3)	40 (14.5)	0	6 (14)	1 (<1%)	40 (14%)
Neutrophil count decreased	0	35 (12.7)	1 (2)	17 (39)	0	35 (13%)
Peripheral sensory neuropathy	0	35 (12.7)	0	5 (11)	0	35 (13%)
Hypothyroidism	24 (8.2)	1 (0.4)	5 (11)	0	23 (8%)	1 (<1%)
Hyperthyroidism	12 (4.1)	1 (0.4)			12 (4%)	1 (<1%)
Pneumonitis	8 (2.7)	0			8 (3%)	0
Infusion reactions	5 (1.7)	13 (4.7)			5 (2%)	13 (5%)
Hepatitis	4 (1.4)	0			4 (1%)	0
Hypophysitis	4 (1.4)	0			4 (1%)	0
Colitis	3 (1.0)	4 (1.4)			3 (1%)	4 (1%)
Severe skin reactions	1 (0.3)	1 (0.4)			1 (<1%)	1 (<1%)
Type 1 diabetes	1 (0.3)	0			1 (<1%)	0
Pancreatitis	0	1 (0.4)			0	1 (<1%)
Adrenal insufficiency	1 (0.3)	0				
Treatment-related AE			28 (60)	42 (96)		
Grades 3–5			5 (11)	28 (64)		
Led to discontinuation			1 (2)	6 (14)		
Led to death			0	2 (5)		
White blood cell count decreased			1 (2	13 (30)		
Aspartate aminotransferase			0	5 (11)		

**Table 2:** Baseline characteristics of the included studies.

**Study arms**		**Number of patients in each group**		**Age (yr**)		**Sex (n**)				**Other baseline diseases**		**Duration of following up**	
Intervention	Control	Pembrolizumab	Paclitaxel	Pembrolizumab	Paclitaxel	Pembrolizumab		Paclitaxel					
						Female	Male	Female	Male	Pembrolizumab	Paclitaxel	Pembrolizumab	Paclitaxel
Pembrolizumab	paclitaxel	296	296	62.5	60	94	202	88	208	Gastrectomy 45 (15.2)	Gastrectomy 51 (17.2)	2 yr	2 yr
Pembrolizumab	paclitaxel	47	47	61	61	15	32	10	37	Gastrectomy 24(51) Gastric ulceration 8 (17)	Gastrectomy 22 (47) Gastric ulceration 11 (23)	4 yr	4 yr
Pembrolizumab	paclitaxel	296	296	62.5	60	94	202	88	208	Gastrectomy 45 (15%)	Gastrectomy 51 (17%)	7·9 mo	7·9 mo
Pembrolizumab	paclitaxel	218	202									7.9 mo	7.9 mo

### 3.3. Risk of bias and quality of evidence

**Figure 2.**
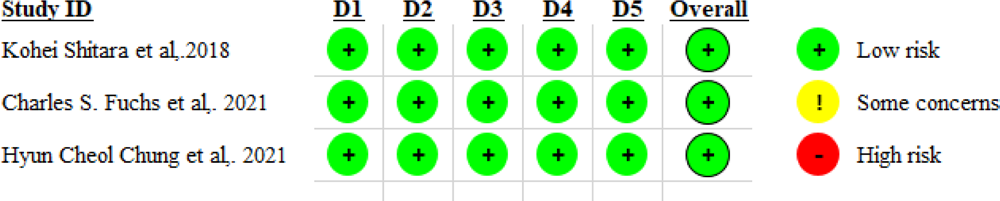
Risk of bias of the included studies in the meta-analysis.

According to Cochrane’s risk of bias tool, the quality of the included trials was good (low risk). We demonstrated the summary of the risk of bias in Fig. 2.

### 3.4. Objective response

**Figure 3.**
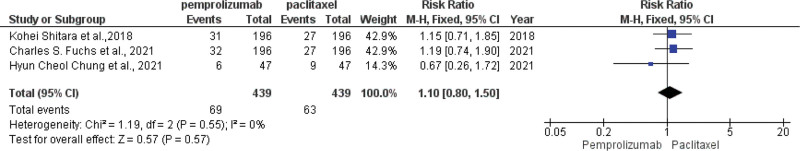
Forest plot of the comparison between pembrolizumab and paclitaxel regarding rate of objective response.

**Figure 4.**
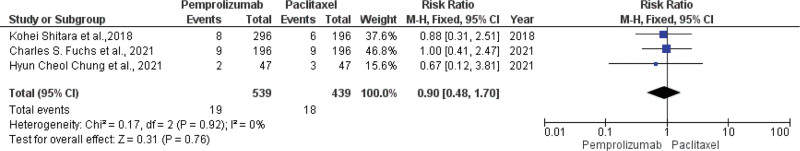
Funnel plot of the comparison between pembrolizumab and paclitaxel regarding rate of objective response.

The overall analysis showed no statistically significant difference between pembrolizumab and paclitaxel regarding rate of objective response (relative risk (RR) = 1.10, 95% CI = 0.80–1.50, *P*-value = .57). We observed no heterogeneity among studies (*P* = .55, *I*² = 0%), Figs. 3 and 4.

### 3.5. Complete response

**Figure 5.**
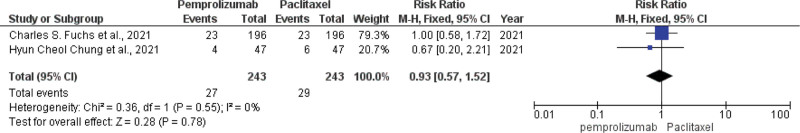
Forest plot of the comparison between pembrolizumab and paclitaxel regarding rate of complete response.

**Figure 6.**
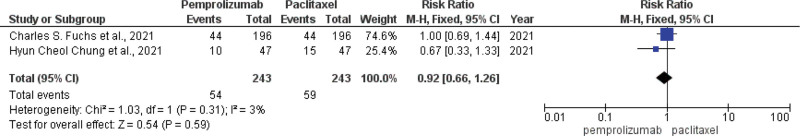
Funnel plot of the comparison between pembrolizumab and paclitaxel regarding rate of complete response.

The overall analysis showed no statistically significant difference between pembrolizumab and paclitaxel regarding rate of complete response (RR = 0.90, 95% CI = 0.48–1.70, *P*-value = .76). We observed no heterogeneity among studies (*P* = .92, *I²* = 0%), Figs. 5 and 6.

### 3.6. Partial response

**Figure 7.**
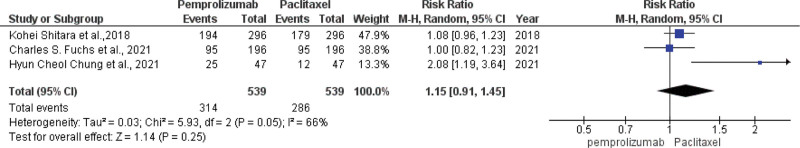
Forest plot of the comparison between pembrolizumab and paclitaxel regarding rate of partial response.

**Figure 8.**
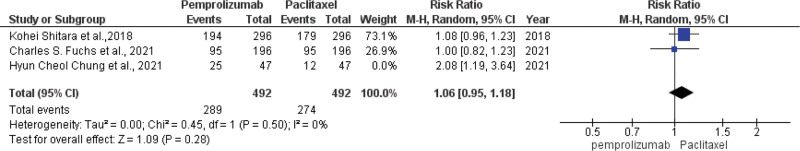
Funnel plot of the comparison between pembrolizumab and paclitaxel regarding rate of partial response.

The overall analysis showed no statistically significant difference between pembrolizumab and paclitaxel regarding rate of partial response (RR = 0.93, 95% CI = 0.57–1.52, *P*-value = .78). We observed no heterogeneity among studies (*P* = .55, *I*² = 0%), Figs. 7 and 8.

### 3.7. Stable disease

**Figure 9.**
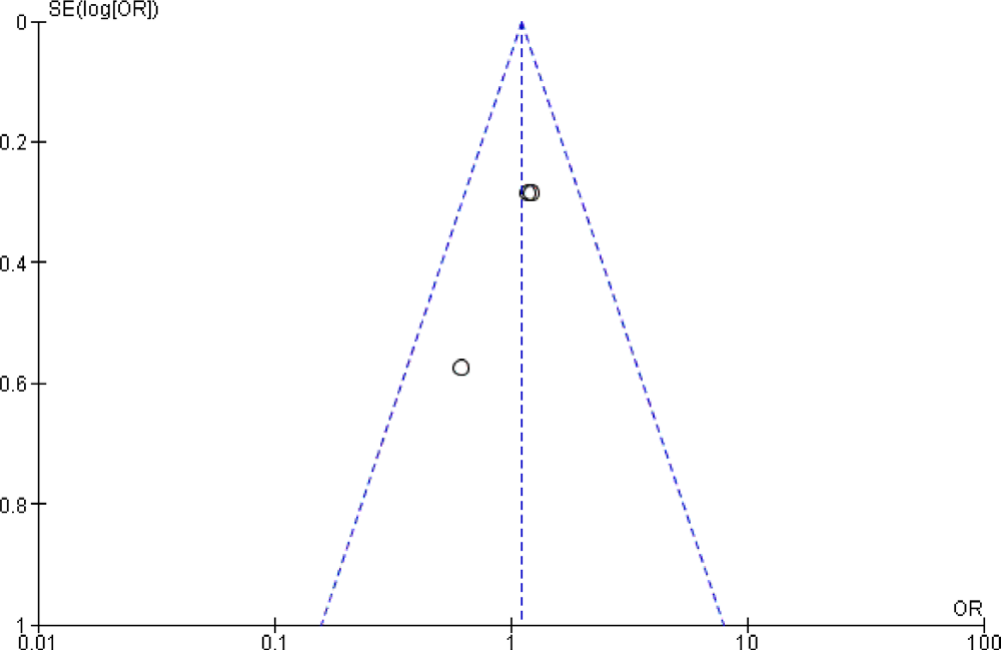
Forest plot of the comparison between pembrolizumab and paclitaxel regarding rate of stable disease.

**Figure 10.**
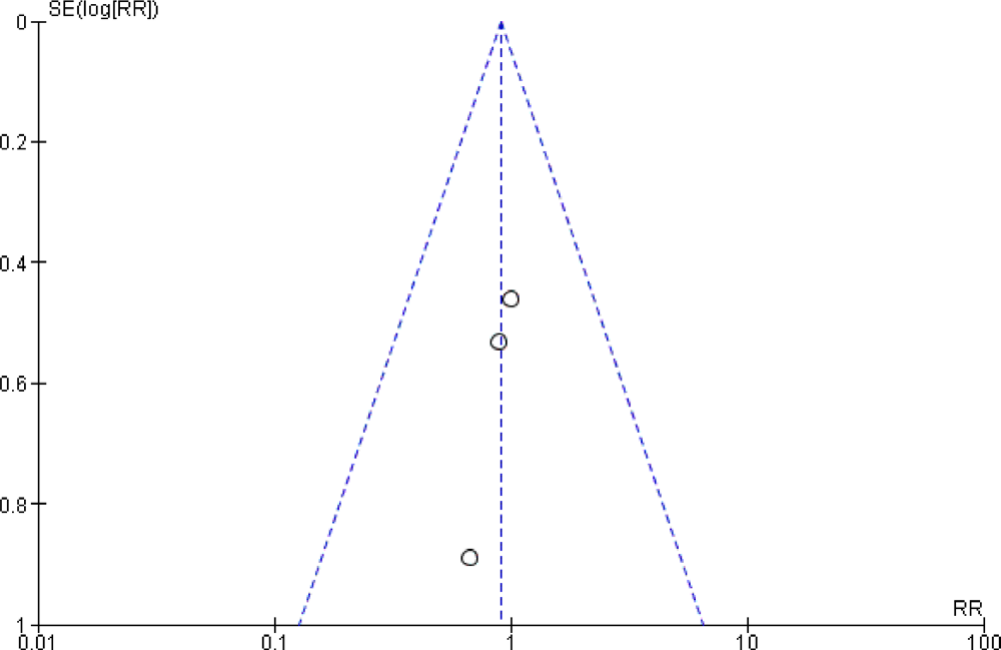
Funnel plot of the comparison between pembrolizumab and paclitaxel regarding rate of stable disease.

The overall analysis showed no statistically significant difference between pembrolizumab and paclitaxel regarding rate of stable disease (RR = 0.92, 95% CI = 0.66–1.26, *P*-value = .59). We observed no heterogeneity among studies (*P* = .31, *I*² = 3%), Figs. 9 and 10.

### 3.8. Progressive disease

**Figure 11.**
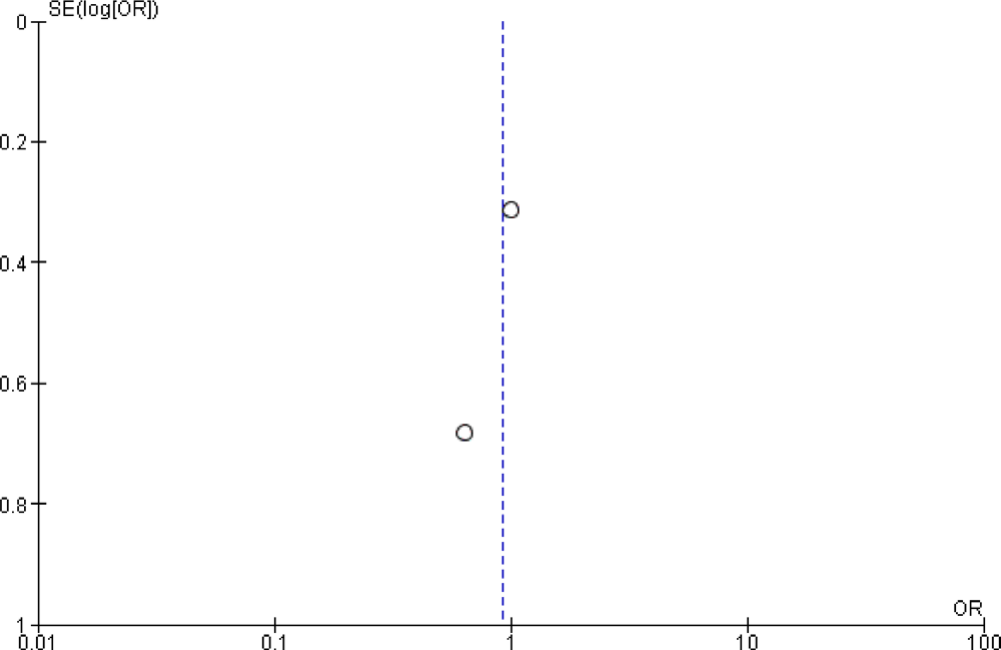
Forest plot of the comparison between pembrolizumab and paclitaxel regarding rate of progressive disease.

**Figure 12.**
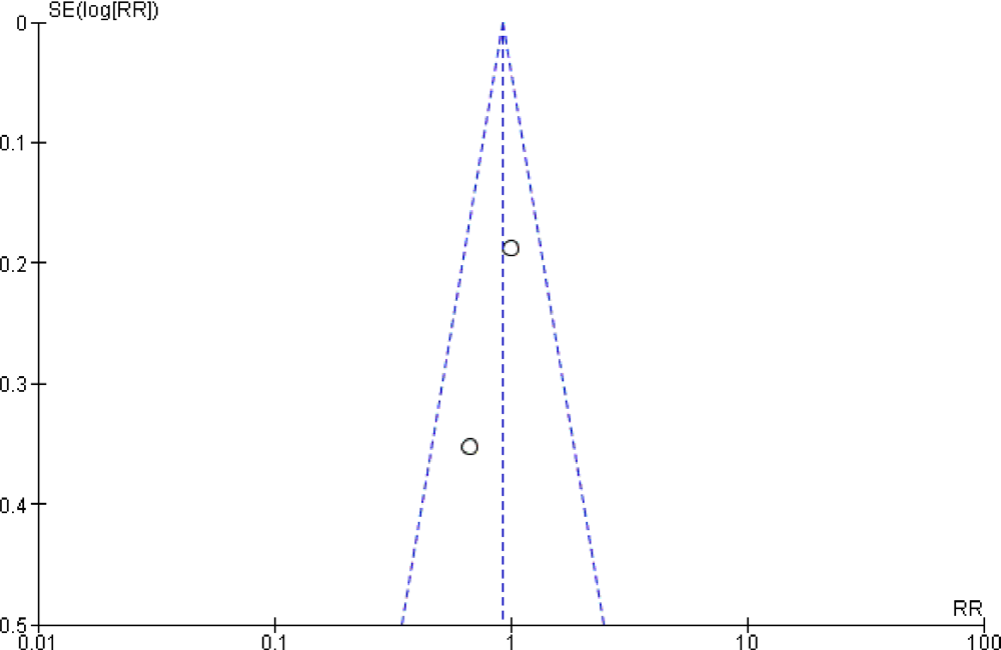
Forest plot of the comparison between pembrolizumab and paclitaxel regarding rate of progressive disease after solving heterogeneity.

**Figure 13.**
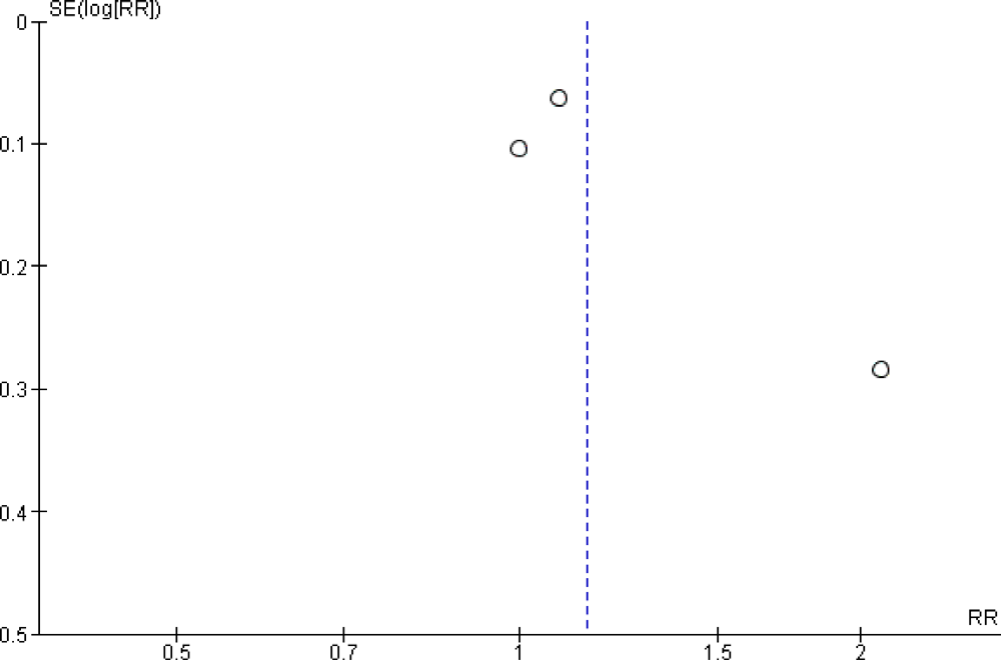
Forest plot of the comparison between pembrolizumab and paclitaxel regarding rate of progressive disease.

The overall analysis showed no statistically significant difference between pembrolizumab and paclitaxel regarding rate of progressive disease (RR = 1.15, 95% CI = 0.91–1.45, *P*-value = .25). We observed a significant heterogeneity among studies (*P* = .05, *I*² = 66%), Fig. 11. We performed leave-one-out test by removing (Chung 2021) study and the heterogeneity was solved (*P = *.50, *I*² = 0%), and the result remained non-significant (RR = 1.06, 95% CI = 0.95–1.18, *P*-value = .28), Figs. 12 and 13.

## 4. Discussion

### 4.1. Introduction

The prevalence of gastroesophageal junction cancer, an uncommon but sometimes fatal illness, has increased as a public health concern in recent years. The absence of consistent categorization criteria has traditionally made it difficult to diagnose this condition. Pembrolizumab can be used safely in different lines of treatment of advanced gastric cancer or gastroesophageal cancer.^[13–16]^ It can be used in advanced gastric or gastroesophageal cancer that is recurrent or metastatic, expresses PD-1, and progresses after at least 2 previous lines of treatment.^[16]^ Pembrolizumab is also approved to be used in solid tumors with metastatic or unresectable microsatellite instability, mismatch repair deficiency, or with a high mutation burden (> 10 mut/MB) with progression after previous treatment and when other alternative treatments are unsatisfactory.^[17]^ Paclitaxel is an antimitotic agent that targets the microtubules inhibiting the occurrence of mitosis by disturbing the mitotic spindles,^[19,20]^ which inhibit cell division and represent the most important step in tumor replication and growth. Paclitaxel can be used as a monotherapy or combination in the second line of treatment of metastatic and recurrent gastric and gastroesophageal cancer. Paclitaxel, combined with ramucirumab, is considered standard in the second-line therapy of advanced cancer, which progresses after initial first-line therapy.

### 4.2. Summary of results

Our study showed no statistical difference between Pembrolizumab and Paclitaxel regarding the rate of objective response, complete and partial, nor the rate of stable disease or progressive disease. This can be explained by the fact that the OS benefit from pembrolizumab is related to the density of PD-L1 expression in the tumor,^[21]^ which may be higher in the tumor cells of the study showed their special effect, and even the mutational burden can determine the response to the second-line therapy using pembrolizumab.^[18]^ The time to respond is similar between therapies, so the objective response rate suggests that clinicians may consider either therapy for patients with advanced gastroesophageal cancer. Comparing the 2 treatments showed no difference in complete or partial response, indicating the similar safety of both drugs. Regarding the disease progression, both pembrolizumab and paclitaxel can be used without a significant effect in esophageal cancer treatment.

### 4.3. Comparison with other studies

Our meta-analysis is the first to compare pembrolizumab and paclitaxel to get the best evidence for gastroesophageal cancer treatment. Multiple trials compared pembrolizumab and paclitaxel in treating gastric cancer after failure of first-line treatment. KEYNOTE-061 study showed that using paclitaxel alone and paclitaxel with ramucirumab showed the superiority of the combination over monotherapy^[22]^ and pembrolizumab is superior in safety while paclitaxel is superior in OS and progression-free survival.^[23]^

### 4.4. Strength limitations

This is the first meta-analysis to look into the effectiveness and response of Pembrolizumab in comparison with Paclitaxel for treating Gastro-Esophageal Junction Cancer. However, the study should be considered in light of several limitations. The first limitation is that the number of studies is not considered big due to the lack of research comparing the 2 drugs; however, the 3 included RCTs had enough sample size (N = 1231 patients). The second limitation is that the study protocol was not registered, which introduces potential bias to the review and does not align with Cochrane guidance.

## 5. Conclusion

As shown in the findings of our meta-analysis, pembrolizumab and paclitaxel do not vary in terms of the rate at which they induce an objective response. Since the duration of response is comparable for both treatments, the objective response rate implies that clinicians may consider using either therapy for patients with advanced gastroesophageal cancer. Clinicians may also consider using both therapies for patients with advanced gastric cancer.

## Author contributions

All authors read and approved the final manuscript.

**Conceptualization:** Sarya Swed.

**Data collection, literature search, and manuscript preparation:** Sarya Swed, Nour Shaheen, Nesreen Elsayed Talat, Samah S. Rozan, Rehab Diab, Karam R. Motawea, Hidar Alibrahim, Mhd Kutaiba Albuni, Elias Battikh, Bisher Sawaf, Sheikh Shoib.

**Final editing:** Abdulqadir J. Nashwan and Wael Hafez.

## Supplementary Material


