# Supplementary material for: Pembrolizumab versus paclitaxel for previously treated, advanced gastro-esophageal junction cancer: A systematic review and meta-analysis of randomized clinical trials
Source: Medicine (Baltimore). 2022 Dec 2;101(48):e31940. doi: 10.1097/MD.0000000000031940 (PMC9726310; doi:10.1097/MD.0000000000031940)
Supplement: Supplementary file 1 [file medi-101-e31940-s001.pdf]

Supplementary files

PubMed search strategy

(("pembrolizumab" [Supplementary Concept]) **AND**

"Paclitaxel"[Mesh]) OR ("Albumin-Bound Paclitaxel"[Mesh] OR "paclitaxel poliglumex" [Supplementary Concept] OR "cyclotriphosphazene-paclitaxel conjugate" [Supplementary Concept] OR "docosahexaenoyl-paclitaxel" [Supplementary Concept] OR "paclitaxel 2'-ethylcarbonate" [Supplementary Concept] OR "130-nm albumin-bound paclitaxel" [Supplementary Concept] OR "2'-(valyl-leucyl-lysyl-PABC)paclitaxel" [Supplementary Concept] OR "paclitaxel-transferrin" [Supplementary Concept] OR "poly(ethylene glycol)-conjugated paclitaxel-2'-glycinate" [Supplementary Concept] OR "N-(2-hydroxypropyl)methacrylamide copolymer-paclitaxel conjugate" [Supplementary Concept] OR "poly(gamma-glutamylglutamine)paclitaxel" [Supplementary Concept] OR "paclitaxel-Angiopep-2 conjugate" [Supplementary Concept] OR "paclitaxel-EC-1 conjugate" [Supplementary Concept] OR "2'-paclitaxel methyl 2-glucopyranosyl succinate" [Supplementary Concept] OR "10-succinyl paclitaxel" [Supplementary Concept] OR "7-(3-methyl-3-nitrosothiobutyryl)paclitaxel" [Supplementary Concept] OR "7-(5'-Biotinylamidopropanoyl)paclitaxel" [Supplementary Concept])

AND ("Esophagogastric Junction"[Mesh]) AND "All MeSH Categories"[Mesh]

<u>Other databases search strategies</u>

(("Pembrolizumab "[Mesh]) **AND** (("paclitaxel *"[Mesh]) **AND**

(("gastroesophageal*"[Mesh]) AND (("cancer*"[Mesh]).
